# Evaluation of surgical outcome of Jack vertebral dilator kyphoplasty for osteoporotic vertebral compression fracture—clinical experience of 218 cases

**DOI:** 10.1186/s13018-016-0371-4

**Published:** 2016-04-30

**Authors:** Jin Fan, Yimin Shen, Ning Zhang, Yongxin Ren, Weihua Cai, Lipeng Yu, Naiqing Wu, Guoyong Yin

**Affiliations:** Department of Orthopaedic Surgery, The First Affiliated Hospital of Nanjing Medical University, Nanjing, Jiangsu 210029 China; Department of Emergency Surgery, The Second Affiliated Hospital of Soochow University, Suzhou, Jiangsu 215000 China

**Keywords:** Fracture, Thoracolumbar vertebra, Osteoporosis, Kyphoplasty, Jack vertebral dilator

## Abstract

**Background:**

Osteoporotic vertebral compression fracture is a serious complication of osteoporosis. Various vertebral kyphoplasty surgeries, which have their own unique features, are commonly used for osteoporotic vertebral compression fracture. Based on the anatomic property of the thoracolumbar vertebral pedicle that its horizontal diameter is twice that of the vertical diameter, we designed Jack vertebral dilator for better restoration of the vertebral height by manipulating the mechanical force.

**Methods:**

A total of 218 patients (236 vertebrae) with osteoporotic vertebral compression fracture were treated with Jack vertebral dilator. Surgery was successfully completed in all cases, and all the 218 patients were followed up for an average of 14.2 months (range 3 to 30 months).

**Results:**

Bone cement leakage occurred in 12 cases, but no symptoms were reported. No other complications were noticed. The VAS scores were 8.2 ± 1.3, 1.7 ± 0.9, and 1.8 ± 0.8 and the ODI was 78.2 ± 13.3 %, 18.5 ± 7.3 %, and 20.9 ± 6.8 % before surgery and 1 week after surgery and at the final follow-up, respectively. The anterior vertebral body height was 19.3 ± 3.2, 25.1 ± 2.6, and 24.9 ± 2.6 mm and the central vertebral body height was 18.7 ± 3.0, 24.8 ± 3.0, and 24.5 ± 2.9 mm before surgery and 1 week after surgery and at the final follow-up, respectively. Cobb angle was 16.2° ± 6.6°, 8.1° ± 5.6°, and 8.5° ± 5.6° before surgery and 1 week after surgery and at the final follow-up, respectively.

**Conclusions:**

Jack vertebral dilator kyphoplasty for osteoporotic vertebral compression fracture is safe, feasible, and effective and has the prospect of further broad application in the future.

**Electronic supplementary material:**

The online version of this article (doi:10.1186/s13018-016-0371-4) contains supplementary material, which is available to authorized users.

## Background

Vertebral compression fracture is a serious complication of osteoporosis and may greatly compromise the quality of life in the elderly; in severe cases, it could be life threatening. Recommended treatment for osteoporotic vertebral fracture includes activity modification, analgesic medications, and back muscle exercise. However, patients often become unable to tolerate activities of daily living and may require bed rest to control pain. This frequently leads to prolonged immobilization, resulting in further losses of bone mass and vertebral height, aggravating back pain and deformity. The elderly suffering from vertebral compression fracture succumb to various complications arising from prolonged immobilization and the mortality rate could reach as high as 23 to 34 % [13].

Hallberg et al. [10] and Johnell et al. [12] showed that conservative treatment for osteoporosis has a poor efficacy, and the 5-year mortality is higher than that for hip fracture. Internal fixation can be used as conventional therapy but may result in surgical failure because vertebral pedicle screw can be easily pulled out. The current major method for osteoporotic vertebral fracture is percutaneous vertebroplasty and percutaneous kyphoplasty. Percutaneous vertebroplasty for relieving pain associated with vertebral angioma was first described in 1987. Thereafter, percutaneous vertebroplasty has been extensively used for the treatment of pain associated with osteoporotic vertebral fracture and vertebral fracture caused by osteolytic malignant cancer [9]. However, percutaneous vertebroplasty cannot restore the lost vertebral height and has a high leakage rate of bone cement up to 40.3 % [11, 17].With the development of minimally invasive surgery, percutaneous kyphoplasty can partially restore the lost vertebral height and reduce cement leakage rate and has been gradually replacing percutaneous kyphoplasty as the preferred method for osteoporotic vertebral compression fracture [8, 16, 24]. Percutaneous balloon kyphoplasty is the most extensively used form of percutaneous kyphoplasty [3, 4, 25], but clinical evidence indicates that the vertebral height is restored only 2.9 mm on average, approximately one third of the lost vertebral height, and Cobb angle is corrected only by 3.4° on average [20]. Bone cement leakage causes severe complications in the lungs and kidney and may lead to brain embolus and even cause death [2, 19]. Despite percutaneous balloon kyphoplasty is associated with a reduced bone cement leakage rate (8.6 %) compared with that of percutaneous vertebroplasty, the bone cement leakage rate is still unacceptably high.

For better restoration of the vertebral height, correction of kyphosis, and reduction of bone cement leakage, new surgical equipments are being developed and evaluated [22, 26]. Based on the anatomic property of the thoracolumbar vertebral pedicle that its horizontal diameter is twice that of the vertical diameter [6, 18], we designed Jack vertebral dilator for better restoration of the vertebral height by manipulating the mechanical force [21]. The clinical results of Jack vertebral dilator kyphoplasty compared with those of percutaneous balloon kyphoplasty revealed reduced bone cement leakage rate. To further observe its efficacy, we retrospectively reviewed the clinical outcome of 218 patients with osteoporotic vertebral compression fracture, who were treated with Jack vertebral dilator from October 2006 to September 2013.

## Methods

The Regional Ethical Review Board in The First Affiliated Hospital of Nanjing Medical University approved the study (date of approval 11 February 2007). All patients gave informed written consent before inclusion in the study. We retrospectively reviewed the clinical data of 218 patients with osteoporotic vertebral compression fracture who received kyphoplasty with the Jack vertebral dilator at the Department of Orthopaedic Surgery, the First Affiliated Hospital, Nanjing Medical University, Nanjing, China from October 2006 to September 2013. A patient was considered eligible for inclusion in the study if (1) he or she had a vertebral bone mineral density (BMD) of *T* < −2.5D, (2) the subject suffered from vertebral fracture within the past 3 months, (3) the vertebral fracture was confirmed by X-ray examination or CT scan, (4) the posterior wall of the vertebral body was largely intact, (5) the signal was low on T1WI or high in T2WI or STIR in MRI, (6) back pain was associated with the fractured vertebral body, (7) there was no neurological deficits, (8) there was no severe heart, brain, and lung problems; and (9) there was no contraindication for surgery such as infection and coagulation disorders.

### The Jack vertebral dilator

**Fig. 1:**
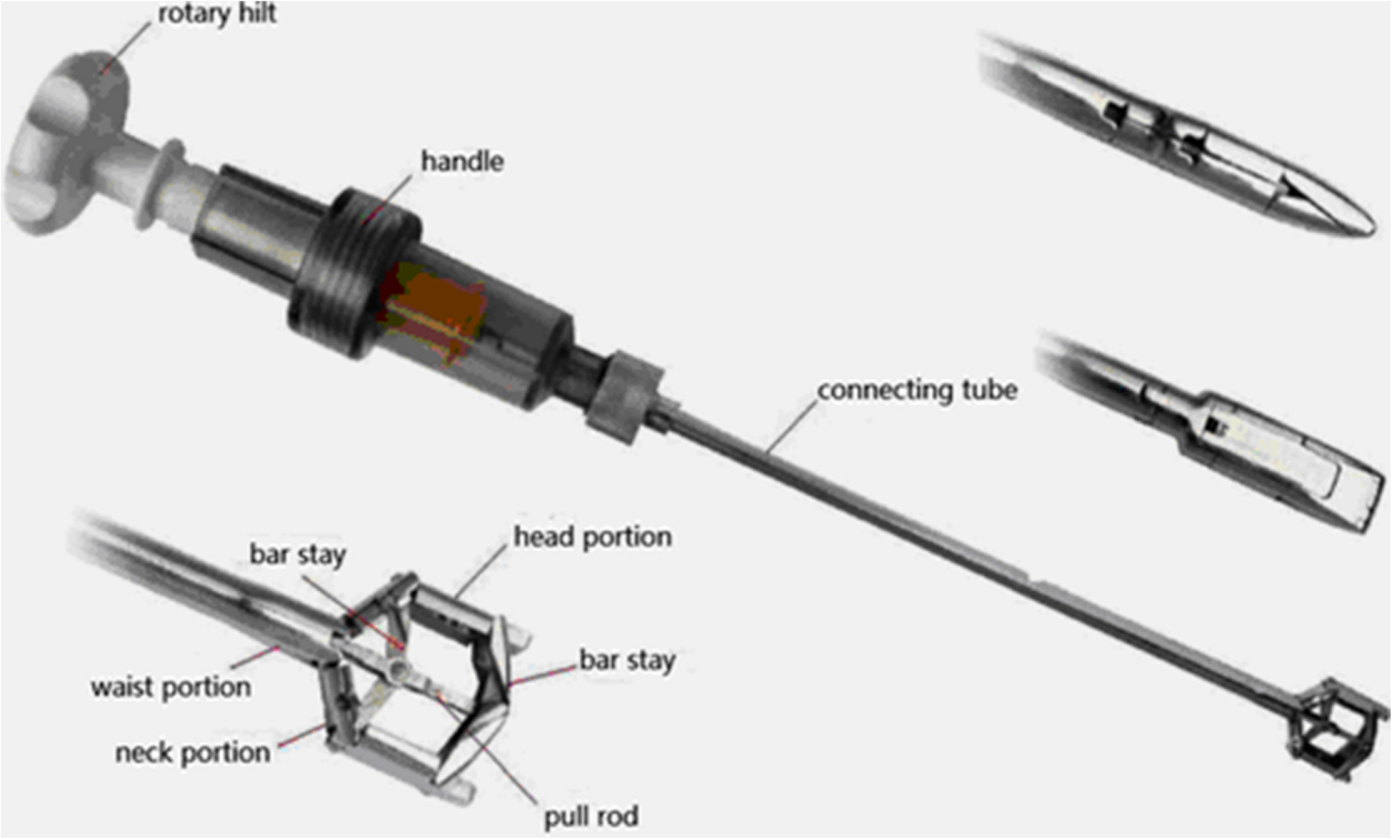
The Jack vertebral dilator consists of a rotary hilt, a handle, and a connecting tube and the head. There are two bar stays, one proximal and the other distal in the head portion. Inside the dilator is a pull rod; and when the pull rod is drawn backward and proximally or pushed forward and distally, it opens or closes the dilator head in a parallel fashion. When the dilator head is completely closed, the proximal and distal bar stay is hid in the inner notch along the head and neck and bar stays. During Jack vertebral dilator kyphoplasty, the generation of a pull force produces vertical tension that helps restore the vertebral height. When the dilator is removed, a cavity is formed, which can be filled in with bone cerement to restore the correct spine position

Base on the previous studies, we designed the Jack vertebral dilator (China patent NO: ZL200920036189.4). As shown in Fig. 1, it consists of a rotary hilt, a handle, and a connecting tube and the head. There are two bar stays, one proximal and the other distal in the head portion. Inside the dilator is a pull rod and when the pull rod is drawn backward and proximally or pushed forward and distally, it opens or closes the dilator head in a parallel fashion. When the dilator head is completely closed, the proximal and distal bar stay is hid in the inner notch along the head and neck and bar stays. When the dilator closes, the thickness and width of the front end of the dilator are 4.8 and 8 mm, respectively, for a small vertebral dilator (suitable for T10 to L1) and 5.3 and 9 mm, respectively, for a large vertebral dilator (suitable for L2 to L5). As the rotary hilt is turned clockwise or counterclockwise, the pull rod moves proximally to generate a push or pull force. During Jack vertebral dilator kyphoplasty, the generation of a pull force produces vertical tension that helps restore the vertebral height. When the dilator is removed, a cavity is formed, which can be filled in with bone cerement to restore the correct spine position.

### Surgical procedure

**Fig. 2:**
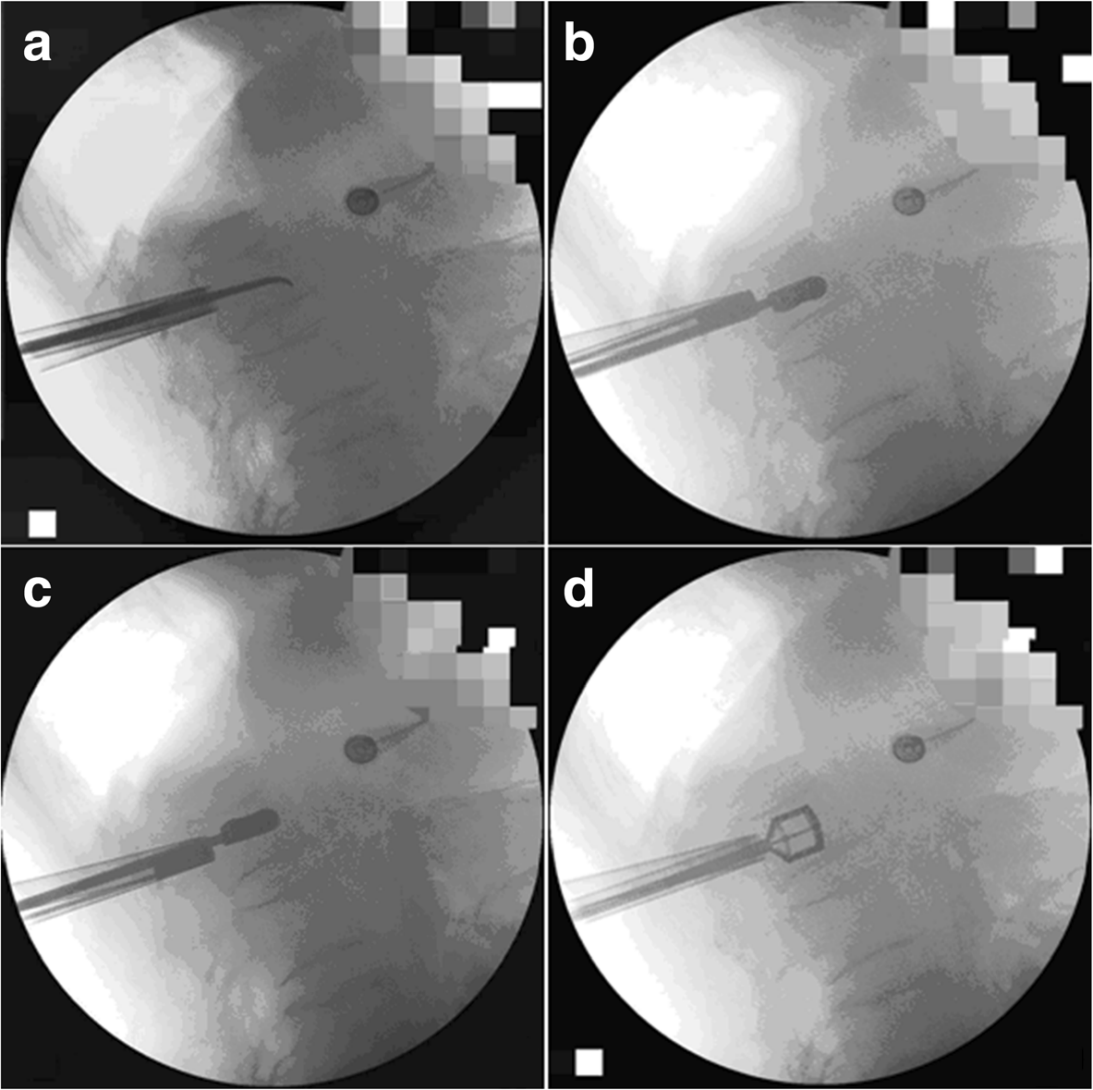
Fluoroscopic graphs taken during Jack vertebral dilator kyphoplasty. **a** Acquisition of tissue specimens for pathological examination during the kyphoplasty; **b** presetting the surgical path inferiorly within the vertebral body; **c** presetting the surgical path superiorly within the vertebral body; **d** the Jack vertebral dilator is fully expanded in the vertebral body

The patient was placed prostrate. The bilateral vertebral pedicle shadows appeared symmetrical and were equidistant from the spinous process under fluoroscopic guidance. The puncture needle was entered at 3 o’clock on the right and 9 o’clock on the left from the vertebral pedicle shadow and reached the posterior wall when advanced to the inner edge of the vertebral pedicle shadow. An annular and then oval working cannula was advanced to the posterior edge of the vertebral body, and a biopsy forceps or a curette was passed through the cannula to obtain tissue specimens from the bone tissue within the vertebral body for pathological examination (Fig. 2a). A dissector that had an identical size and shape to the dilator was advanced along the working cannula inside the vertebral body and a tunnel of certain height in the vertebral body was preset using a specially made locator (Fig. 2b, c). The vertebral dilator was placed horizontally in the mid 2/3 of the vertebral body and rotated 90° clockwise. The wider part of the head of the dilator faced towards the superior and inferior endplate. The dilator handle was rotated clockwise, and the dilator was opened at the superior and inferior endplate and was nearly parallel (Fig. 2d). The dilator rotary hilt was then turned counterclockwise 90°, and the dilator was removed. PMMA was prepared and filled into the cavity in the vertebral body via the cement delivery tube. Filling was stopped if the cement overflowed or when the cement was filled to 1 to 2 mm from the posterior edge of the vertebral body. The patient lay flat on the bed for at least 1 h after the procedure. The surgical procedure was performed by two equally experienced orthopedic surgeons (Additional file 1).

### Patient evaluation

Anterior posterior and lateral radiographs of the thoracic and lumbar spine were taken before and 1 week after surgery and at the final follow-up. The anterior, central, and posterior heights of each of the 13 vertebral bodies from T4 to L4 were measured using an electronic caliper to determine the vertebral body height. The anterior height was determined by measuring the length between the most antero-superior point of the superior endplate and the most antero-inferior point of the inferior endplate of the fractured vertebral body. The mid or central body height was determined by measuring the distance between the midpoint of a line connecting the most antero-inferior and postero-inferior points of the inferior endplate and that of the most antero-superior and postero-superior points of the superior endplate of the fractured vertebral body. The posterior vertebral body height was measured between the most postero-superior point of the superior endplate and the most postero-inferior point of the inferior endplates of the fractured vertebral body. Cobb angle was determined in the lateral view by measuring the angle formed between a line drawn parallel to the superior endplate of one vertebra above the index vertebra and a line drawn parallel to the inferior endplate of the vertebra one level below the index vertebra. Vertebral fractures were considered to be present if at least one of three height measurements taken from along the length of the same vertebra was decreased by more than 20 % compared with the height of the nearest uncompressed vertebral body. BMD values were measured by dual-energy X-ray absorptiometry using QDR-2000 (Hologic Inc., Waltham, MA) at the lumbar spine. BMD was automatically calculated from the bone area (square centimeters) and bone mineral content (grams) and expressed absolutely in grams per square centimeter. The T-score is the number of SD by which a given measurement differs from the mean for a normal young adult reference population. The intensity of the patient’s pain was assessed by the visual analog scale (VAS 0 to 10, 0 representing no pain and 10 representing worst pain ever experienced) at rest on the day before the procedure. The pain was reassessed at 1 week after surgery and at the final follow-up. Furthermore, the effect of back pain on the daily quality of life was determined by the Oswestry Disability Index (ODI) [7].

### Statistical analysis

Data were expressed as $\overline{x}\pm \mathrm{s}$ and analyzed using the SPSS 17.0 software. Paired *t* tests were used for comparison of data prior to operation and 1 week post surgery and at the last follow-up. *P* < 0.01 was considered statistically significant.

## Results

### Patient demographic and fracture characteristics

Two hundred and eighteen patients with osteoporotic thoracolumbar vertebral compression fracture met the eligibility criteria for Jack vertebral dilator kyphoplasty. The demographic and fracture characteristics are shown in Table 1. They included 180 female and 38 male patients with their age ranging from 47 to 86 years (mean, 68.2 years). The duration of back pain ranged from 0.1 to 1.5 months (mean, 0.6 month). The preoperative lumbar vertebral BMD T-score was −2.9D to −2.5D. T1WI signal was decreased while T2WI or STIR signal was increased in the fractured vertebra, which matched the vertebral segment with spinous process pain and tenderness in the patients. Acute or subacute vertebral fracture was confirmed in all the subjects and the fractured vertebral body was identified to be T10 (2 vertebrae), T11 (16 vertebrae), T12 (62 vertebrae), Ll (82 vertebrae), L2 (32 vertebrae), L3 (20 vertebrae), L4 (20 vertebrae), and L5 (2 vertebrae). The anterior and central vertebral body heights were compressed by 2.2 to 13.6 mm (mean, 8.7 mm) and 5.2 to 13.8 mm (mean 8.9 mm), respectively. The posterior wall of the vertebral body remained largely intact. No neurological deficits were observed.

**Table 1:** The demographic and baseline characteristics of the patients

Variable	DKP
Age in years (range)	68.2 (47–86)
Gender (%)	
Male	38
Female	180
Bone cement volume in milliliter (SD)	5.51 (0.75)
Vertebral bodies (%)	
T10	2 (0.8)
T11	16 (6.8)
T12	62 (26.3)
L1	82 (34.7)
L2	32 (13.6)
L3	20 (8.5)
L4	20 (8.5)
L5	2 (0.8)

Data are displayed as mean ± standard deviation or number (percentage)

### Surgical outcome

Jack vertebral dilator kyphoplasty was successfully performed in all the subjects, including a total of 236 vertebrae. The mean operative time was 78 ± 7.5 min. Four to 8 mL of bone cement (mean volume, 5.51 ± 0.75 mL) was filled in the vertebral body on both sides. The patients started activities 2 to 3 days after bed rest. Calcitriol was used routinely postoperatively. Alendronate was prescribed when patients started activities. The mean hospital stay was 8 ± 0.5 days. There was no report of mechanical failure. No gross leakage out of the vertebral limits was observed using real-time imaging and X-rays at surgery and the subsequent follow-up. No signs of root irritation or neurological deficit were observed throughout the surgery or subsequent to the procedure. No infection or signs of embolism were noted.

The patients were followed up for 14.2 months (range 3 to 30 months). There was no loss to follow-up. The anterior vertebral body height was 19.3 ± 3.2 mm before surgery, which increased to 25.1 ± 2.6 and 24.9 ± 2.6 mm at 1 week post surgery and at the final follow-up, respectively (*P* < 0.01 versus before surgery) (Table 2). The anterior fractured vertebral body height was restored to 84.7 % of normal height. Furthermore, the central vertebral body height was 18.7 ± 3.0 mm before surgery, which increased to 24.8 ± 3.0 and 24.5 ± 2.9 mm at 1 week post surgery and at the final follow-up (*P* < 0.01 versus before surgery) (Table 2). The central fractured vertebral body height was restored to 84.6 % of normal height. Additionally, Cobb angle in these patients was 16.2° ± 6.6° before surgery, which decreased to 8.1° ± 5.6° and 8.5° ± 5.6° at 1 week post surgery and at the final follow-up (*P* < 0.01 versus before surgery). Cobb angle was corrected 7.7° ± 3.4° on average.

**Table 2:** The anterior and central vertebral body height and Cobb angle in patients with osteoporotic vertebral compression fracture $\left(\overline{x}\pm s,n=236\right)$

Before surgery	Anterior vertebral body weight (mm) 19.3 ± 3.2	Mid vertebral body height (mm) 18.7 ± 3.0	Cobb angle (°) 16.2 ± 6.6
One week post surgery	25.1 ± 2.6^*^	24.8 ± 3.0^*^	8.1 ± 5.6
Final follow-up	24.9 ± 2.6^**^	24.5 ± 2.9^**^	8.5 ± 5.6

^*^ *P* < 0.01 versus the preoperative data; ^**^ *P* > 0.05 versus 1 week post surgery

On admission, the patients reported a mean VAS score of 8.2 ± 1.3. The VAS score decreased to 1.7 ± 0.9 and 1.8 ± 0.8 at 1 week post surgery and at the final follow-up (*P* < 0.01 versus before surgery) (Table 3). In addition, the ODI of these patients was 78.2 ± 13.3 before surgery, which decreased to 18.5 ± 7.3 and 20.9 ± 6.8 at 1 week post surgery and at the final follow-up (*P* < 0.01 versus before surgery).

**Table 3:** Visual analog scale (VAS) scores and Oswestry Disability Index (ODI) in patients with osteoporotic vertebral compression fracture (*x* ± *s*, *n* = 236)

Before surgery	VAS 8.2 ± 1.3	ODI (%) 78.2 ± 13.3
One week post surgery	1.7 ± 0.9^#^	18.5 ± 7.3^#^
Final follow-up	1.8 ± 0.8*	20.9 ± 6.8*

^#^ means that *P* < 0.01 versus the preoperative data; *P > 0.05 versus 1 week post surgery

### Typical case

**Fig. 3:**
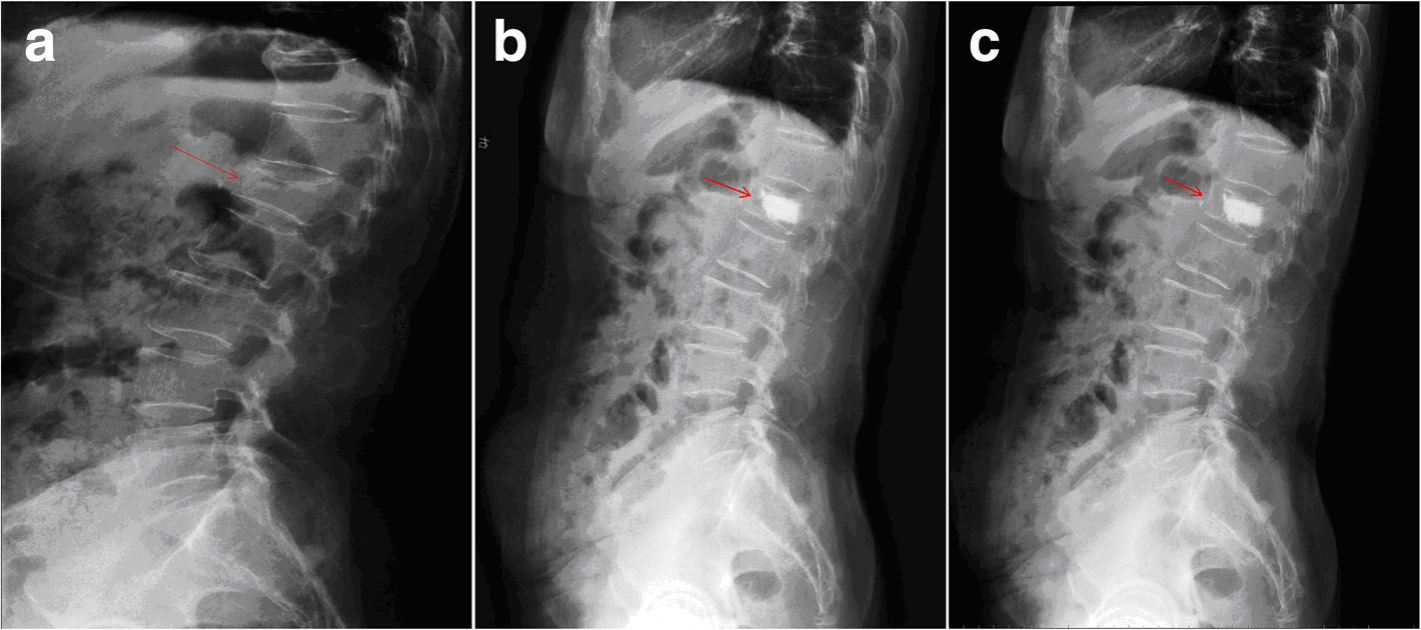
A patient with a BMD T-score. **a** Preoperative X-ray examination revealed vertebral compression fracture at L1; the anterior vertebral body height was 23.3 mm and compressed 17.6 % and the central vertebral body height was 17.5 mm and compressed 38.2 %. Cobb angle was 18.2°. **b** Roenterography 1 week post surgery revealed that the anterior vertebral body height was 27 mm and was restored to 84.4 % of the original height and the central vertebral body height was 25.0 mm and was restored to 88.2 % of the original height. Cobb angle was 15.9°. Bone cement filling was adequate. **c** Roenterography at the final follow-up (8 months post surgery) revealed that the anterior vertebral body height was 26.8 mm and was restored to 83.8 % of the original height and the central vertebral body height was 24.4 mm and was restored to 86.1 % of the original height. Cobb angle was 16°. Bone cement filling was adequate

As shown in Fig. 3, a 51-year-old female patient who suffered from vertebral compression fracture at L1 was treated with Jack vertebral dilator.

### Complications

The major complication in the current series is bone cement leakage, including a small leakage on the lateral side of the vertebral body in 8 cases and in the intervertebral space in 4 cases. The leakage rate was 5.1 %, but no symptoms were reported. No neurological deficits were observed and there was no case of pulmonary embolism. Fracture of the vertebral body adjacent to the index vertebra occurred in 4 cases during follow-up, a second Jack vertebral dilator kyphoplasty was performed, and the patients recovered satisfactorily.

## Discussion

Because the distal pull rod of the Jack vertebral dilator is relatively far from the distal end of the head portion, early Jack vertebral dilator kyphoplasty has a smaller expansion force. To increase the expansion force of the head portion, we have improved the design of the Jack vertebral dilator (not shown in this article). The follow-up study showed that Jack vertebral dilator kyphoplasty for osteoporotic vertebral compression fracture can well restore vertebral height, correct kyphosis, and reduce bone cement leakage rate. Additionally, it maintains the corrected spine position for a longer period of time postoperatively. The preoperative VAS scores were 8.2 ± 1.3, which were significantly reduced to 1.8 ± 0.8 at the final follow-up. Furthermore, the ODI went from 78.2 ± 13.3 % preoperatively to 20.9 ± 6.8 % at the final follow-up. These findings suggest that Jack vertebral dilator kyphoplasty effectively relieves pain and improves the quality of life of patients with osteoporotic vertebral compression fracture.

The design of Jack vertebral dilator takes full advantage of the anatomic properties of the thoracolumbar vertebral pedicle, and the orifice dilating tunnel of the vertebral pedicle maximally utilizes the vertebral pedicle height. The surgical path in Jack vertebral dilator kyphoplasty is wider than that of balloon kyphoplasty, and the bone cement delivery tube in Jack vertebral dilator kyphoplasty has an inner diameter of 3.9 mm and an outer diameter of 4.5 mm while that in balloon kyphoplasty has an inner diameter of 2.8 mm and an outer diameter of 3.4 mm; hence, the cross-sectional area of the surgical path in Jack vertebral dilator kyphoplasty is twice that of balloon kyphoplasty. Bone cement enters the vertebral body as a viscous paste. Loeffel et al. [15] and Baround et al. [1] also found that bone cement at an appropriate viscosity reduces bone cement leakage. Therefore, bone cement in its viscous paste form can effectively reduce the risk of bone cement leakage and is safer. During Jack vertebral dilator kyphoplasty, bone cement in its paste form is filled into the cavity left by the dilator and infiltrates the adjacent osteoporotic bone tissues, which forms a small amount of pseudopods and enters the intertrabecular space, thus preventing bone cement paste from becoming displaced in the vertebral body. Complete filling of the vertebral body with bone cement is not actively pursued. This practice thus further reduces the risk of bone cement leakage and minimizes the effect of bone cement on the blood and lymph circulation in the bone trabeculae, ensuring an uninterrupted supply of nutrients to osteocytes within the trabeculae. The bone cement leakage rate in the current series is only 5.1 %, which is markedly lower than 8 % seen in balloon kyphoplasty [20].

Moreover, the elliptic surgical path in Jack vertebral dilator kyphoplasty has sufficient space to allow the passage of a biopsy forceps or curette to obtain biopsy specimens from within the vertebral body. This helps determine the cause of vertebral body fracture, for example, osteoporotic vertebral body fracture or pathological vertebral body fracture caused by osteolytic malignant cancer, which provides important evidence for the ultimate diagnosis and subsequent treatment of vertebral body fractures. A tunnel of certain height in the vertebral body in the elliptic surgical path can be preset using a specially made dissector. When the head of the dilator expands and the upward vertical expansion force is constant, the angle between the pull rod and the handle is very small during the early stage of head opening. By the parallelogram law, the pull force for the pull rod in this situation is very large and may be 6 to 8 times as large as the vertical expansion force, which predispose the dilator to damages. When the angle between the pull rod and the handle reaches above 30° and if the head portion is to achieve the same effective expansion force, the pull force for the pull rod is 1.7 times less than the vertical expansion force. Therefore, presetting a tunnel of certain height (12–13 mm for a large dilator and 9–11 mm for a small dilator) can noticeably reduce the resistance during the initial phase of expansion and minimize the force on the pull rod and bar stay.

CT scan of the thoracolumbar spine and two-dimensional reconstruction are routinely carried out before Jack vertebral dilator kyphoplasty. The height and width of the vertebral pedicle are also determined. These measurements help a surgeon determine the size of vertebral dilator. Unipedicular injection of bone cement cannot be carried out by enlarging the angle of the puncture needle and advancing the needle over the midline of the vertebral body. Steinmann et al. [23] found that unipedicular kyphoplasty did not differ significantly in restoring vertebral body height and reconstruction of vertebral body strength and stiffness compared with bipedicular kyphoplasty. However, Libeschner et al. [14] found that single-sided load transfer may cause spine instability, which positively correlated with the filling volume of bone cement. Unipedicular injection of bone cement compared with bipedicular injection more noticeably causes relative motion of the vertebral body from the treated side to the contralateral side. With increasing filling volume of bone cement, scoliosis from unipedicular injection will become more apparent. It has been observed that scoliosis is more severe in patients with overfilled bone cement compared with untreated patients. Chung et al. [5] also believe that bipedicular kyphoplasty is superior to unipedicular kyphoplasty in biomechanical properties and restoration of vertebral heights.

## Conclusions

The current study demonstrates that Jack vertebral dilator kyphoplasty for osteoporotic vertebral compression fracture is safe, feasible, and effective and has the prospect of further broad application in the future.

## Additional file

Additional file 1: Surgical procedure. (MOV 129672 kb)
